# Tissue Engineering Techniques for Induced Pluripotent Stem Cell Derived Three-Dimensional Cardiac Constructs

**DOI:** 10.1089/ten.teb.2021.0088

**Published:** 2022-08-08

**Authors:** Tori Salem, Zachary Frankman, Jared M. Churko

**Affiliations:** ^1^ Department of Cellular and Molecular Medicine, University of Arizona Health Sciences, Tucson, Arizona, USA.; ^2^ Department of Biomedical Engineering, University of Arizona Health Sciences, Tucson, Arizona, USA.

**Keywords:** organoids, EHTs, iPSCs, cardiac tissue, tissue engineering

## Abstract

Recent developments in applied developmental physiology have provided well-defined methodologies for producing human stem cell derived cardiomyocytes. The cardiomyocytes produced have become commonplace as cardiac physiology research models. Accessibility has also allowed for the development of tissue engineered human heart constructs for drug screening, surgical intervention, and investigating cardiac pathogenesis. However, cardiac tissue engineering is an interdisciplinary field that involves complex engineering and physiological concepts, which limits its accessibility. Our review provides a readable, broad reaching, and thorough discussion of major factors to consider for the development of cardiovascular tissues from stem cell derived cardiomyocytes. In this study, our review will examine important considerations in undertaking a cardiovascular tissue engineering project and will present, interpret, and summarize some of the recent advancements in this field. Throughout, we review different forms of tissue engineered constructs, a discussion on cardiomyocyte sources, and an in-depth discussion of the fabrication and maturation procedures for tissue engineered heart constructs.

**Impact statement:**

With advancements in cardiac differentiation protocols, the production of human induced pluripotent stem cell derived cardiomyocytes is becoming cost effective and routine in the laboratory setting. Monolayer based culture methods are rapidly being replaced by three-dimensional (3D) tissue engineered constructs, which are more representative of the heart geometry. In the review presented, we delve into important concepts and tissue engineering principles that should be considered when generating 3D cardiac constructs, interpreting data acquired from, and embarking on a 3D cardiac tissue-based research project.

## Introduction

Heart failure rates are increasing,^1^ and more than 650,000 deaths are associated with cardiovascular disease each year in the United States.^2^ Given that heart failure rates are rising, investigating heart failure is a major research focus.^3^ The multitude of factors influencing the heart presents a significant challenge in the development of treatments for cardiovascular disease and the study of cardiac pathogenesis. For example, emotional stress,^4^ kidney functionality,^5^ and gut microbiota^6^ have been identified as significant factors that can modulate heart function. With an improved scientific understanding of factors influencing the human heart, heart failure can be combated through treatment, prevention, and even tissue engineering based surgical therapy.

Developing a deeper scientific understanding of the human heart through direct experimentation is not feasible due to ethical concerns.^7^ In addition, human based research can be difficult to reproduce and can be costly.^8^ The difficulty in reproducibility originates from the high variability in patient lifestyle, age, comedication, comorbidity, and from costs associated with obtaining a high sample size.^9^ Furthermore, only half of clinical trials are tested for reproducibility, and half of those tested were shown to be reproducible.^10^

**FIG. 1.**
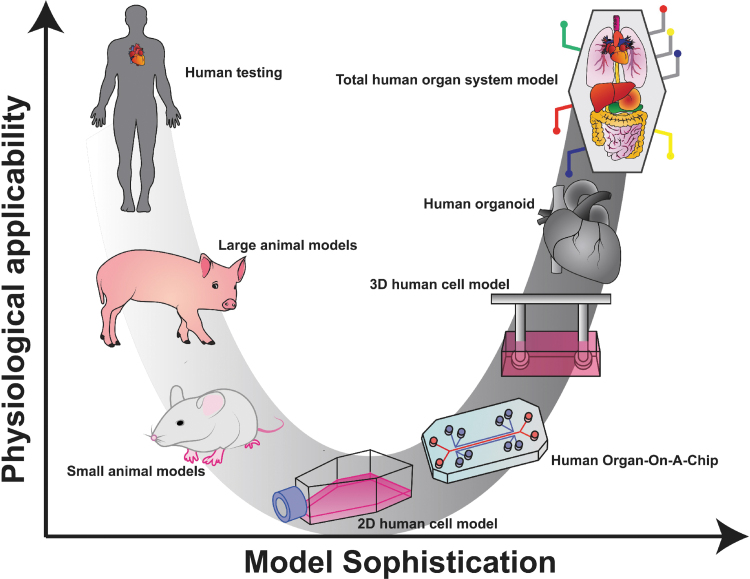
Biological models differ in applicability and sophistication. Biological models for the human body exist on a continuum of physiological applicability and model sophistication. Animal models are more sophisticated as they spare a human subject and are applicable in that they demonstrate the systemic effect of a treatment. Isolated cells, organ-on-a-chip, and 3D human cell models are more sophisticated approaches that allow for continuous observation and greater control over the stimuli provided to the heart cells but are incapable of recreating the influence of tissue-level and total systemic responses. Human heart organoids recreate tissue-level responses, and the idealized goal of a total organ system model would be capable of realizing total systemic responses. 2D, two-dimensional; 3D, three-dimensional. Color images are available online.

The most common method for investigating cardiac physiology is through the use of preclinical trials on biological models, with computational models arising recently as an inexpensive alternative.^11^ These biological models come in three general forms—animal models and cell- and tissue-scale engineered heart constructs. Such models are advantageous compared to human models in several ways, as they allow researchers to induce disease states synthetically or exert an increased degree of control over the physiology of the system **(**Fig. 1**)**. Animal models have historically been used as biological models to parse out individual mechanisms in the heart. However, animal models imperfectly approximate human physiology due to the multitude of physiological differences between human and animal hearts.^12,13^ For example, the resting heart rate in a mouse is ∼500 to 700 beats per minute (bpm).^14–18^ The resting heart rate of an adult human is between 60 and 90 bpm.^19–22^ The considerable heart rate differences between mice and humans make the study of heart diseases such as arrhythmogenic cardiomyopathy difficult. The more expensive and logistically complicated canine and porcine models possess fewer physiological differences from human hearts, but these differences are non-negligible. Aside from having nearly double the resting heart rate of humans, both pigs and dogs are quadrupeds, giving them altered valvular anatomy and subtracting much of the gravitational components of venal drainage found in humans.^12^ Furthermore, the porcine cardiac electrical activity is conducted primarily by specialized cardiac muscle cells, whereas the human heart possesses fewer neural ganglion cell bodies and primarily propagates its action potentials myogenically.^23^ The differences between human and animal models become even starker at the transcriptomic level.^24,25^ Succinctly, even very accurate animal models are insufficient to negate the need for extensive human clinical trials.

Because of the drawbacks of animal models for heart research, biologically engineered organ constructs are an attractive alternative. Cell-scale heart models have been under investigation since the 1990s and can be used to recreate the heart on a series of microfluidic chips.^26^ Microfluidic chips represent an “Organ-On-A-Chip,” which act as a platform to rapidly screen drugs for their impact on human heart cell contraction and metabolism.^27^ The concept of microfluidic chips is promising because alterations in human cardiomyocyte functionality can be detected in a high throughput manner with a relatively lower cost.^26,28^ However, microscale and monolayer-based systems do not allow for spatially separated three-dimensional (3D) coculturing or the direct observation of cardiac remodeling, both of which are significant contributors to cardiac tissue behavior.^29^ Tissue-scale heart constructs are a larger and more reagent intensive version of cell-scale heart constructs, but with the added benefit of allowing for 3D cell culture, observation of cardiac remodeling, and the coculture of multiple cell types in physiologically relevant spatial distribution.^30–32^ Furthermore, tissue-scale heart constructs can be used in surgical interventions for heart failure as heart tissue patches.^33^ Both cell- and tissue-scale heart models are under intensive investigation and have the potential to allow for relevant and high-throughput experimentation on human heart tissue to rapidly investigate disease pathogenesis and develop useful tissue engineered heart constructs.

Our review will outline the motivation for research using tissue engineered cardiac constructs, detail what considerations and techniques go into engineering cardiac constructs, and expound on the current discoveries and projects in cardiac tissue engineering.

## Engineered Cardiac Constructs

**FIG. 2.**
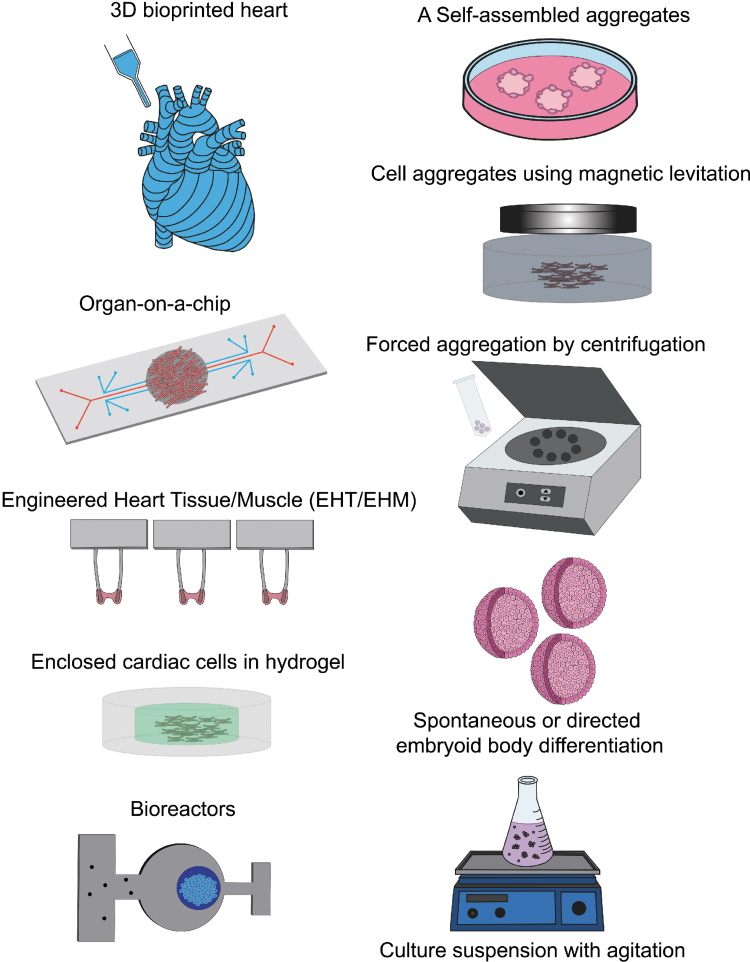
Three-dimensional induced pluripotent stem cell-derived cardiac constructs. A variety of 3D cardiac constructs have been developed to study cardiac physiology and to mimic the microenvironment of the heart. 3D bioprinted cardiac constructs promote higher spatial and anatomical accuracy with a mixture of various matrices and cardiac cells. Organ-on-a-chip and other microfluidic chips mimic complex human cardiac tissue at a miniaturized scale. EHTs/EHMs are optimized to measure the contractile force of cardiomyocytes mixed in a matrix and suspended between two posts. Hydrogels exhibit tissue-like properties to model the physiologically relevant cardiac microenvironment. Bioreactors mimic the fluid dynamics and nutrient need to assess cardiac constructs. Cardiac constructs can be generated using aggregation methods such as monolayer culture on a low adhesion surface matrix to spontaneously self-aggregate, magnetic levitation, forced aggregation using centrifugation, and spontaneous or directed cardiac differentiation in embryoid body form which can be produced at a larger scale using culture suspension with agitation (magnetic- or shaker based). Color images are available online.

Various cardiac constructs have been developed throughout the years (Fig. 2), and these constructs have been shown to produce more mature and physiologically accurate (addition of protein and collagen matrices)^34^ cardiac models compared to monolayer-based cultures. Specifically, 3D cardiac constructs have been reported to possess an enhanced cardiac ion channel density, faster upstroke velocity, increased catecholamine response,^35^ and matured membranous structure formation (T-tubule-like structures).^36^ These 3D cardiac constructs are assembled by various methods and may contain various cardiac cell types and matrices. A promising platform to assemble cardiac constructs is 3D bioprinting.^37,38^ Deposition of cardiac cell types and “bioinks” (permanent or dissolvable) spatially controls the construct architecture, cell type interactions formed, and the physiological relevance of the cardiac construct.^39,40^ This method has shown promise for use in anatomical studies, drug discovery/screening, and pathophysiological modeling.^41–45^

Another method to generate 3D cardiac constructs is through self-aggregation. If cardiac constructs are generated by differentiating stem cells into cardiomyocytes, stem cells cultured in suspension will self-aggregate to form embryoid bodies (EBs). EBs can be expanding in suspension using a shaking platform or by magnetic agitation and directed to differentiation into beating cardiomyocyte clusters through growth factors and small molecule treatment further described below. Furthermore, cardiac cells cultured on a low adhering matrix will spontaneously self-aggregate.^46–49^ In addition, 3D cardiac constructs can also be generated using directed aggregation methods. Cells which have taken up magnetic nanoparticles can be forced to aggregate when a magnet is added below a culture well.^50–52^ Cells can also be forced to aggregate by being centrifuged into cone-shaped microwells.^53^

Various vessels support the culture environment of cardiac constructs. Aggregates enclosed within bioreactors which control the microenvironment of cells with a continuous flow of nutrient-rich media^54^ are shown to be more robust, have enhanced viability, and more uniform cell distribution and alignment.^55^ In addition, an overall improvement in cell distribution and morphology, cardiac protein expression, and tissue organization is seen with the use of perfusion in cardiac cell aggregates.^56–58^ Aggregates can also be molded into specific matrices or hydrogel to mimic the extracellular matrix environment of cardiac cells.^59,60^ Crosslinked 3D networks composed of hydrophilic polymers mimic the soft and flexible structures and water content of native tissues, which promote a more physiologically accurate cardiac model.^61^ Another vessel with a defined purpose involves the integration of microfluidic technology with living cells on slides to generate “organ-on-a-chip” systems. Organ-on-a-chip systems are focused on studying organ-specific interactions and replacing animal drug testing models.^62^

Finally, cardiac constructs such as engineered heart tissues or engineered heart muscles (EHTs/EHMs)^63,64^ have been developed with a focus on understanding cardiac contraction parameters and will serve to demonstrate a system that integrates many of the topics discussed in our article. The EHT system uses a convex mold to form molten agarose into 1 cm long rectangular cavities in each well of a 24-well plate. Two flexible posts are then suspended vertically in each well, and the cavity is filled with cardiomyocytes in a crosslinking matrix. After gelating, the resultant heart tissue is found to be strung between the two flexible posts and can be removed from the mold and placed in media, where it develops. Electrodes can be inserted on either side of the tissue to electrically pace the construct. Gravity tensions the tissue, promoting sarcomere alignment and causing contractions to pull the beams together. The resultant construct's contraction amplitude can be quantified into force units through the use of the two flexible beams attached to each end of the construct. Because the posts’ Young's modulus (E, units N/m^2^), length (L, units m), and cross-sectional moment of inertia (I, units m^4^) are known, video analysis that can measure the axial postdeflection (δ, units m) can be used to find the contraction force (F, units N or kg·m/s^2^) using the simple equation^65^ $F=\frac{3EI\delta }{L^{3}}$. The equation is one of the most widely used 3D tissue models that incorporates electrical and passive mechanical stimulation with contraction force transduction, with many applications having been explored since its inception in 2010.^63^ The system has also been modified extensively by investigators to include a variety of unique features, such as the addition of piezoelectric actuator for active mechanical stimulation, and a modular add-on that allows for the incremental increase of passive mechanical stimulation of the postbeams^66,67^ (Table 1).

**Table 1.** Studies Utilizing the Engineered Heart Tissue Platform

Verification of EHT platform	A review of the EHT platform and notable indications of accuracy in recreating realistic behavior of cardiomyocytes in monoculture. This review includes confirmation of accurate inotropic reactivity, orientation and structure, functionality, and cell morphology.^239^
	A verification that iPSC-derived cardiomyocytes, when adequately matured in the EHT system, have a physiologically accurate positive force frequency relationship.^223^
	A confirmation that EHT matured cardiomyocytes in monoculture develop a resting membrane potential similar to the right or left ventricle *in vivo*. Outward potassium ion channel concentration in iPSC-CM-EHTs was also found to be comparable to human mature cardiomyocytes. iPSC-CM-EHTs did not have functioning acetylcholine activated potassium channels, indicating that they do not have a right ventricular phenotype. Action potential waveform indicates a left ventricular phenotype.^240^
Cell signal and drug screening performed	Evidence that miR-24 controls smooth muscle cell proliferation and vascularization, shown using the EHT platform.^241^
	Evidence that blocking miR-140-3p stops deterioration in cardiomyocytes under stress, shown using the EHT platform.^242^
	Evidence that myosin binding protein C reduces the deterioration of cardiomyocytes under stress, shown using the EHT platform.^243^
Attempts at increasing physiological accuracy	A review of attempts to increase the physiological accuracy of tissue engineered heart constructs. Many of the constructs were not created using the EHT platform, but with functionally identical platforms. Briefly, mixing fibroblasts and endothelial cells increased contractility, and engineered heart constructs can be induced to pathological conditions when given the same stimuli as found *in vivo*.^244^
	A mixture of epicardial cells and cardiomyocytes improves contractility of the EHT system.^245^
Mechanical stimulation	A novel rack that has an inflexible steel rod that replaces one of the beams on each EHT beam pair. The steel rod is attached to a piezoelectric actuator which deflects the inflexible beam to stretch the tissue. This setup was also modified to fit a much larger EHT in a six-well plate. This setup improved contractility.^67^
	A magnetic-based system that uses one immovable post and one magnetic post. The magnetic post's stiffness is increased by bringing a magnet closer to it, which resists the cardiomyocyte contraction and increases the “afterload” perceived by the heart. This setup found that afterload tripled the force exerted by the heart.^246^
	A modification of the EHT system that uses both a stiff beam and PDMS inserts that increase the force required to deform the beams. This allows for an analysis of how afterload impacts contractility.^66^
Similar systems	A system that uses independent beam sets instead of a rack of four.^247^
	A system that uses a solitary beam of cardiomyocytes in a custom well.^248^
	A thin filament of cardiomyocytes suspended in a mold.^217^
	A fibrous mesh of heart tissue. Coculture of cardiomyocytes and fibroblasts at a 7:3 ratio optimized contractility.^249^

CM, cardiomyocytes; EHT, engineered heart tissue; iPSC, induced pluripotent stem cell; PDMS, polydimethylsiloxane.

## Fabrication Techniques

Various fabrication techniques exist to generate engineered cardiac constructs. One method, called molding, involves adding cells to a prepolymer solution that is then permitted to crosslink, forming a hydrogel that traps the cells within. Molding creates an immediately cellularized construct, although different protocols and scaffold choices may impact the distribution of cells. For instance, bovine collagen and rat tail collagen set at different rates due to differences in molecular weights, with bovine collagen setting so slowly that cells may settle out before gelation. The use of hydrogels with a suspension of cells requires a concave mold to shape the tissue, into which the uncrosslinked hydrogel mixture is poured to set. After the hydrogel gelates, the tissue is then removed from the concave mold, revealing a conjugate construct.

Another method for fabricating cellularized heart constructs called seeding involves fabricating an extracellular matrix in a particular shape, which is later populated with cells.^68^ The method of seeding may involve the use of decellularized cardiac tissue due to its physiological relevance or the generation of completely synthetic matrices using electrospraying,^69^ electrowriting,^70^ or electrospinning^71^ techniques due to their excellent material properties. The precise placement of aligned fibers that these techniques provide is particularly useful in cardiac tissue engineering, as aligned fibers have been shown to promote cardiomyocyte alignment, unidirectional action potentials along the fiber grain, and improved intracellular electrical coupling through the major gap junction protein, connexin 43.^72^ In addition, these techniques allow for a composite matrix, which is to say a scaffold with unique material properties in localized regions, akin to a skeleton. The scaffold allows for the implementation of desired material parameters on a tissue scale without compromising on material composition ideal on a cell scale.^73^ These unique attributes make electrospraying, electrospinning, and electrowriting excellent for specialized cardiac tissue engineering projects.

Another method of construct generation is the use of 3D printing. The three most well established forms of cellular 3D printing are printers that extrude “bio-ink” in stacked layers,^74^ “bio-ink-jet” printers that squirt tiny droplets of cells in stacked layers using heat or pressure,^75,76^ and the photolithography printers which use light to crosslink a solution containing cells and a photosensitive hydrogel precursor.^77,78^ These techniques allow for complex 3D shapes to be created, with the proper spatial distribution of matrix composition and cell lines. For example, myocardial patches with internal endothelial vascularization have been fabricated in a single process using 3D printing techniques.^79^

## Engineered Cardiac Construct Uses

The predominant use of tissue engineered cardiac constructs is to model the composition and function of the human heart. Therefore, generating tissue engineered constructs as biological models involves the generation of cardiac cells *in vitro* to fabricate human cardiovascular tissues that precisely match the *in vivo* heart. Once constructed, stimuli can be introduced to help understand the mechanisms of disease and cardiac remodeling. The combination of various cell types, extracellular matrices, and soluble factors is necessary to replicate or approximate the heart's native tissue microenvironment.^80^ In tissue engineering, generating a construct that can support various heart proteins in combination with human pluripotent stem cells is advantageous since they can be differentiated into all human resident heart cells.

Another aim of cardiac tissue engineering is to fabricate surgical products for implantation to achieve an improved clinical outcome. The goal involves the study of how to maximize construct functionality and tissue integration. There are several loci of interest in the clinical field, including surgical patches, recellularized hearts, and total heart construction. Concisely, projects focused on the fabrication of surgical projects can be thought of as direct translational research.

**FIG. 3.**
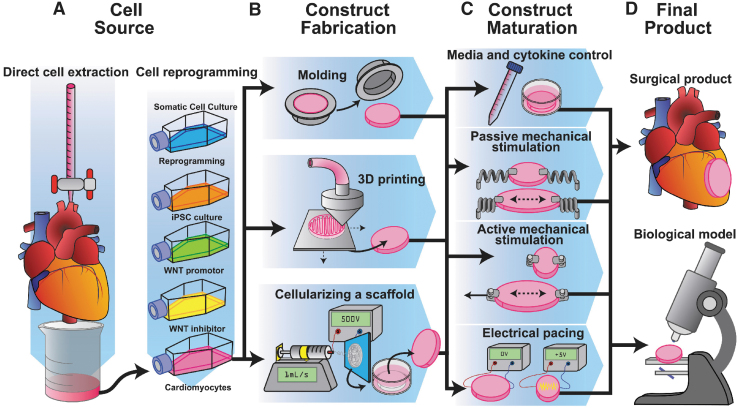
Techniques for cardiovascular tissue engineering. A summary of techniques for cell acquisition, construct fabrication, tissue maintenance, as well as the two major end products. **(A)** Heart cells are either retrieved from extracted heart tissue or generated through stem cell-based differentiations. **(B)** Isolated heart cells are then formed into a 3D construct through a variety of techniques. **(C)** 3D constructs are then matured through chemical, mechanical, and electrical stimulation. **(D)** The finalized product is then used to either model disease and study heart physiology or is used to form a useful heart construct for a patient. Color images are available online.

While there are currently no Food and Drug Administration (FDA) approved tissue engineered surgical products for the heart, stem cell and cardiomyocyte injections have been in use for some time.^81^ However, some animal preclinical trials for tissue engineered heart constructs intended as surgical products are underway^82–85^ **(**Table 2**)**. A detailed review has been written on the topic of heart patches.^86^ The distinction between “surgical product” and “modeling” oriented tissue engineering projects is important to keep in mind, as the end goal of each project informs the decisions made therein **(**Fig. 3**)**. For instance, a project intended to produce a surgical product might make use of anatomically incorrect scaffold geometries such as honeycombs to improve contraction,^87^ with the intent of studying how to maximize force generation for the creation of more effective heart patches. Comparatively, a modeling project might make use of a coculture of cardiomyocytes and endothelial cells to more accurately recreate conditions *in vivo*.^73^

**Table 2.** Projects Intended for Surgical Implantation

Projects for implantation	The “BioVAD”, a pouch made of rat cardiomyocytes which fits over the heart. There was no substantial change in rat heart function after implantation.^250^
	A patch composed of rat neonatal heart cells, which was stitched onto the heart of a living rat with a myocardial infarction. This patch improved QRS amplitude stroke volume.^251^
	A bioreactor that electrically paces myocardial patches derived from rat hearts.^196^
	A honeycomb shaped heart patch to maximize contraction forces, made with rat neonatal cardiomyocytes.^87^

## Engineered Cardiac Construct Components

### Cell source

In a tissue engineered heart construct, cells are the most important component. There are two primary methods of gathering human heart cells—direct extraction from discarded tissue and the generation of cardiomyocytes through stem cell differentiation. Direct extraction yields more mature cardiomyocytes for use as a biological model and is typically performed on either discarded human tissue or from animal tissue.^88–90^ Cardiomyocytes that are extracted have been found to be large, rod shaped, and highly contractile.^88,91^ However, adult human cardiomyocytes are difficult to obtain.

An alternative is to generate human cardiomyocytes from stem cells.^92–96^ Human induced pluripotent stem cells (iPSCs) can be created by expressing four transcription factors into terminally differentiated cells to convert these cells into a pluripotent state.^97^ Once iPSCs have been generated, they can be expanded^98^ and differentiated into nearly any cell type **(**Fig. 3A**)**. Pluripotent stem cells can be induced to differentiate into cardiomyocytes in a variety of ways, most notably through the use of growth factors.^99–101^ A growth factor based cardiac differentiation protocol using bone morphogenetic protein 4 (BMP4), basic fibroblast growth factor (FGF2), and activin is widely used to differentiate cardiomyocytes.^102^ However, a small molecule biphasic Wnt signaling modulation cardiac differentiation method^103–105^ is a fast, efficient, and cost-effective way to generate a high purity of iPSC derived cardiomyocytes. In addition, mechanical cues alone have been shown to differentiate cardiomyocytes through the integrin α and β signaling pathways.^80,106,107^ Several review articles have outlined the various methods used to differentiate and purify cardiomyocytes.^108,109^ One major advantage of iPSCs is the ability to generate stem cell derived cardiomyocytes from patients for use to model their patient-specific phenotype.^110^ Patient-specific phenotype models allow for the generation of virtually unlimited genetically identical cardiomyocytes from specific subjects for an intensive investigation into the exact genetic mechanisms of dysfunction for that phenotype.^103,111^

Cardiac tissues can also be differentiated in a more relevant manner through the use of a combination of mechanical and chemical cues in 3D *in-situ* differentiation, in which stem cells are added into a 3D matrix and provided with chemical signals to induce cardiac differentiation.^112,113^ 3D *in-situ* cardiac differentiation may more accurately recapitulate the signal transduction pathway experienced by stem cells *in utero* and has been shown to produce differentiated tissues with spatially separated cardiac cell types.^49^

### Matrix and composition

**FIG. 4.**
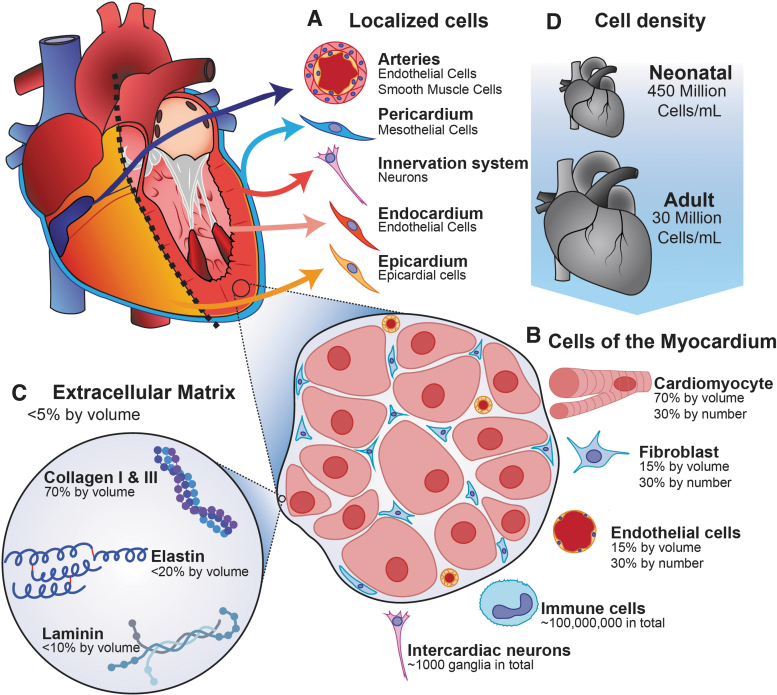
The tissue-level physiology of the human heart. The composition of the heart is varied based on position. **(A)** The heart has various subcomponents, including vasculature, the pericardium, neuronal innervation, and the endocardium. **(C)** Fibroblasts regulate the extracellular matrix of the heart, which is primarily composed of collagen I and III, elastin, and laminin. **(B)** The bulk of the heart is composed of the myocardium which by volume, is primarily composed of cardiomyocytes. The myocardium is heavily vascularized by capillaries, and other cell types are dispersed throughout. **(D)** The cell density of the human heart is highly variable, with neonatal human hearts having more than 10 times the cell density of mature hearts. Color images are available online.

Due to the dynamic and kinetic nature of cardiovascular tissue, the cell density and protein matrix composition are important parameters to consider.^114^ While a hepatocyte might function on a rigid surface, a heart tissue's contraction would be less quantifiable (but not impossible to study^115^) if its force is exerted on a rigid surface. In addition, given the importance of the flexural, compressive, and tensile material properties of the heart, it is important to model the material composition found in the human heart **(**Fig. 4**)**.

Only 2–4% of a healthy human heart's volume is composed of the extracellular matrix.^116^ The matrix found in the human ventricular myocardium is around 70% collagen types I, III, V, and VI,^117^ 80% of which is type I and the remainder of which is primarily type III.^118^ The remaining 30% of the matrix is composed of elastin and dozens of other proteins and glycoproteins (like laminin and fibronectin) found in smaller amounts.^119^ Collagen is largely inert when interacting with cardiomyocytes in monoculture, although there is some amount of interaction between extracellular collagen and surface-bound proteins on the cardiomyocyte.^120^

The cellular composition of the heart is also critical to consider. Seventy percent of the heart's volume is composed of contracting cardiomyocytes, with the remaining 30% composed mostly of fibroblasts and endothelial cells, although all three cell types are found in roughly equal quantities due to fibroblasts and endothelial cells having a much smaller volume.^121^ Cardiac fibroblasts in coculture have been shown to improve cardiomyocyte force production,^122^ and endothelial cells in coculture with cardiomyocytes improve cardiac development, contractility, and rhythmicity by secreting factors such as nitric oxide and neuregulin.^123^ In addition, neurons and immune cells are mixed throughout in low density.^124–126^ In terms of cellular density, the adult heart has on average 28 ± 7.2 million cardiomyocytes/mL, with neonatal hearts possessing 430 ± 72 million cardiomyocytes/mL.^127^

Cardiomyocytes have some ability to remodel their matrix by secreting collagen, but the majority of matrix maintenance is controlled by both fibroblasts and endothelial cells.^128^ There are few (but not zero^120^) interactions between collagen and human cardiomyocytes, making cardiomyocytes in monoculture somewhat ambivalent if collagen was exchanged with another matrix material. Similarly inert but flexible polymers may be used as matrix components in surgical product-based projects. In contrast, there are some notable interactions between the noncardiomyocyte cells in the heart and the extracellular matrix within the cardiac tissue that cannot be overlooked when an accurate recreation of the heart is intended. For example, fibroblasts indirectly influence cardiac function by regulating the extracellular matrix of the heart, which is achieved by fabricating and breaking down the heart's extracellular matrix continuously.^128^ Chemical and physical signals modulate this dynamic balance, and maladaptive signals are the source of some forms of cardiomyopathy. For instance, excessive angiotensin II, aldosterone, and deoxycorticosterone are all signals which promote cardiac fibroblasts to fabricate an excessive amount of collagen, leading to ventricular fibrosis.^118^ Similarly, endothelial cells produce a significant amount of collagen in response to certain physical signals, particularly ventricular overload.^129^ Fibroblasts and endothelial cells also produce matrix metalloproteases (MMPs), which degrade collagen but not necessarily other polymers with different compositions. Therefore, if a consistent and robust model of cardiovascular dysfunction is intended, noncardiomyocyte–cardiomyocyte interactions must be included, necessitating the use of a scaffold composed primarily of collagen or perhaps using a scaffold intended to be wholly replaced with collagen by fibroblasts over time.

Another consideration is the importance of stiffness and viscoelasticity on tissue properties. Fibrosis is a pathogenic state that is the subject of a major field of cardiovascular research, during which collagen stiffens the heart. Stiffness impacts cardiomyocytes in several ways, including their action potential,^130^ metabolic activity,^131^ gene expression, and contractility.^132^ The parameter that signifies the stiffness of an elastic material is the Young's modulus, which describes the amount of force over an area (N/m^2^) required to achieve a certain amount of “stretch” (also known as engineering strain, a unitless ratio), resulting in a unit of N/m^2^, which is equivalent to the unit of Pascals (Pa). A Young's modulus of 9.5 ± 1.5 kPa has been observed in decellularized pig hearts along their cellular alignment and 3.2 ± 00.7 kPa perpendicular to cellular alignment.^133^ The strain at which the decellularized porcine extracellular matrix failed was 25%.^134^ Despite this, cellularized heart tissue was found to be much less stiff.^133^ Destructive material testing on the human heart is less common, as both human and porcine hearts have very similar matrix compositions.^135^ Nevertheless, noninvasive ultrasonic techniques show that healthy young human hearts have a Young's modulus of 2.5 kPa, increasing to 6 kPa by the age of 60.^136^ Both of these levels of stiffness can be achieved with a matrix by creating a dense enough scaffold by controlling the concentration (typically 1–10 mg/mL) of matrix polymer in the prematrix solution, although the addition of cells does alter the resulting stiffness by obstructing polymer–polymer interactions and introducing cell–polymer interactions.^137^ Another important consideration is the viscoelasticity of a scaffold, which is the sensitivity of a scaffold's stiffness to the rate at which it is physically manipulated. The viscoelasticity of a material typically is described using a generalized Maxwell model, a differential equation that can calculate the stress experienced by a material when it experiences a certain rate of stretch (strain rate). Several models exist that take viscoelasticity into account.^138^ The viscoelastic properties of collagen and other matrix substances have been quantified as well, which allow for the precise tuning of the mechanical properties of heart tissue constructs to match the kinetic material properties of the heart.^137,139^ Another important note is that many matrix compositions are characterized by their shear modulus (G) and its derivatives the elastic modulus (G′) and the viscous modulus (G″). These shear moduli are used instead of the Young's modulus due to how hard it is to grip and pull on low-density matrices. A commonly used collagen density for tissue engineering is 2 mg/mL, which has an elastic modulus (G′) of 40.12 ± 3.29 Pascals and a viscous modulus (G″) of 3.43 ± 0.33 Pascals at 49% strain.^140^ The material properties of the extracellular matrix of tissue engineered cardiac constructs have a significant impact on construct functionality and gene expression,^141^ most notably through the mitogen activated protein and extracellular signal-regulated kinase (MAPK and ERK) pathways.^142,143^ Specifically, these pathways are modulated by mechanosensitive surface proteins such as Cav1 and β1 integrin,^144^ and they are regulated by the mechanosensitive gene iex-1 (*IER3*).^145^ Through these mechanisms, cardiac tissue reacts to excessive stiffness, leading to functional modification^146^ or apoptosis.^147^ Furthermore, on the tissue scale, very dense matrices slow cardiomyocyte contraction and relaxation by physically dampening movement, which impacts performance.^148^

With these considerations in mind, there are many choices in matrix composition for use in cardiac tissue engineering, with the most common being collagen, fibrin clots, Matrigel and equivalent products, and various other molecularly tailored resorbable polymers (Tables 3 and 4). Technically all these materials are hydrogels, which is to say a cross-linked scaffold in low concentration that absorbs a large amount of fluid, to the extent that 90% or more of the resulting gel is water by volume.

**Table 3.** Biologically Derived Scaffold Solutions for Three-Dimensional Cell Culture

Polymer	Description	Example uses	Considerations
Fibrin	Thrombin and Fibrinogen can be mixed to rapidly form a crosslinked hydrogel.	EHM^122^ Heart patches^252^ EHTs^63^	Not immediately physiologically relevant to the heart's matrix. This material has an RGD charged amino acid sequence that readily allows cells to bind to its surface.
Collagens	Collagen stored under acidic conditions to prevent crosslinking is neutralized, mixed with cells, and heated to crosslink.	EHTs^63^	Physiologically relevant
Matrigel	A combination of proteins, glycoproteins, and cytokines derived from lysed mouse sarcomas, producing a liquefied basement membrane. When heated above 14°C, it crosslinks and forms a hydrogel. Its major components are laminin and collagen IV.	EHTs^63^ Pacemaker cardiomyocytes with vascularization^253^	An imperfectly characterized substance that will vary from batch to batch.
Other biologically derived hydrogels	Hyaluronic acid, gelatin, chitosan, alginate, and dozens of other proteins, glycoproteins, and GAGs have been used to form hybrid biodegradable scaffolds.	Alginate gels^254^ Gelatin gels^255^ Chitosan gels^256^	These materials have tunable characteristics, such as pore size and stiffness, which make them useful for adding unusual properties, including timed biodegradation or extremely low cost.
Biologically modified hydrogels	The extracellular matrix is not perfectly uniform, and certain pathological and healthy conditions lead to postprocessing. Some of these modified proteins have superior properties for tissue engineering.	Glycated collagen with improved stiffness^257^ UV-denatured collagen with improved stiffness^258^	There are many permutations of hydrogel bases and modifications, some of which are highly specialized for specific projects.

EHM, engineered heart muscle.

**Table 4.** Engineered Solutions for Three-Dimensional Cell Culture

Polymer name	Description	Example uses	Considerations
PLGA	A copolymer of lactic and glycolic acid, two monomers with different bond strengths. By changing the ratio of these two components, their hydrolysis rate can be tuned. PLGA has been used to form hard sponges at high densities and flexible hydrogels at low densities.	Electrospinning into filamentous scaffold, forming over dissolvable templates, crosslinking to form hydrogels^259^	Intended as a robust initial matrix to be dissolved and wholly replaced over time. Used clinically in implants.^260^
Poly (glycerol sebacate)	An engineered two-part polymer that has excellent biocompatibility and easily tunable degradation rates and stiffness.	Heart patches^261^	Cells cannot be integrated into the scaffold during the formation process due to high temperatures and vacuum conditions. Cells must infiltrate into the scaffold postformation.
Photosetting hydrogels	PEGDA is an inert and biodegradable polymer that rapidly crosslinks when exposed to UV light and has been used to form 3D printed constructs through photolithographic printing.^262,263^ Other similar photosetting hydrogels exist.^264,265^	Creation of vasculature in engineered cardiac tissue^77^	The field of fabricating 3D printed biological constructs is rapidly evolving, so new and improved photosetting hydrogels are constantly being published.
PEG	A tunable biodegradable and largely inert polymer which is typically used for microscale tissue engineering, although it can be used as a surface treatment or used to fill up a more rigid scaffold.	High throughput microscale cardiac coculture with endothelial and fibroblasts for drug screening.^266^ PEG as a surface treatment.^267^	Typically used for 2D cell culture.
Modified biological hydrogels	Biological hydrogels improved using chemicals or postprocessing techniques.	Highly cross-linked collagen using a synthetic crosslinking compound.^268^	Maintains physiological applicability of the base matrix, but with modified characteristics.

2D, two-dimensional; PEG, polyethylene glycol; PEGDA, poly (ethylene glycol) diacrylate; PLGA, poly-lactic-glycolic-acid.

## Engineered Cardiac Construct Maturation

As components of a highly metabolic, dynamic, and kinetic tissue, cardiomyocytes require a complex combination of culture conditions to mature in a physiologically relevant manner. The maturation has been investigated thoroughly, and the following section will highlight significant protocols that have been used to generate more mature cardiomyocytes. Nevertheless, immature cardiomyocytes have been used as models for embryonic hearts,^149^ and immature cardiomyocytes possess useful properties for surgical products.^150,151^ As such, cardiac tissue maturation protocols must be carefully chosen to suit the intended purpose of the construct.

### Media

Media components influence human induced pluripotent stem cell-derived cardiomyocytes (hiPSC-CMs) maturity, and various media compositions have been published to promote maturation.^152–154^ A common base media for cardiomyocyte culture and maturation is RPMI 1640 media containing the B27 supplement (a supplement containing various peptides, lipids, and cell viability components). However, various small molecules, amino acids, and hormones (dexamethasone, thyroid hormone [T3], and insulin-like growth factor 1 [IGF-1]^152,155–161^) have been identified to promote iPSC-CM maturation **(**Table 5**)**. Given that cardiomyocytes *in vivo* primarily derive their energy from fatty acids,^162^ some studies emphasize the importance of fatty acid supplementation (most notably oleic, linolenic, and lipoic acid^163^) to improve the maturity of iPSC derived cardiomyocytes,^162,163^ and other groups have emphasized that culturing cardiomyocytes using a combination of fatty acids and high glucose concentration leads to a more mature gene expression profile and contraction activity.^164–166^ However, normal blood glucose levels are around 5 mM,^167^ and supraphysiologic glucose levels have been shown to induce adipogenesis and alter cardiomyocyte development.^164–166^ Furthermore, culturing and differentiating human pluripotent stem cells can be costly, and alternative, cost-effective media formulations exist to culture (B8) and differentiate iPSCs into cardiomyocytes (CDM3).^103,168–170^

**Table 5.** Cardiac Cell Maturation Components

Classification	Maturation component	Description
Culture supplement	B27	B27 is a media supplement containing various hormones, lipids, and amino acids that promotes iPSC-CM maintenance and maturation.^269^
	Insulin	Insulin supports the growth and metabolism of myocardial cells.^270^
Hormone	Thyroid hormone (T3)	T3 increases contractile force, calcium release and reuptake, and cardiomyocyte size.^155–157,271^
	Dexamethasone	Dexamethasone, which is a glucocorticoid which enhances electromechanical maturation of iPSC-derived cardiomyocytes.^152^
Growth factor	IGF-1	IGF-1 regulates contractility, metabolism, hypertrophy, and apoptosis in the heart.^155^
Fatty acids	Oleic acid	Fatty acids improve contractile force, metabolism, and function of cardiac cells.^163,271–273^
	Palmitic acid/palmitate	
	Linoleic acid	
	Sodium L-lactate	
Small molecules	Phosphodiesterase inhibitor (IBMX)	The addition of IBMX increases contractile activity and force.^160,161^
	HIF-1α inhibitor (FM19G1)	Increases fatty acid oxidation.^272^
	PPARα agonist (WY-14643)	Facilitates mitochondrial metabolic maturation.^272^
	mTOR inhibitor (Torin1)	Facilitates cardiomyocyte quiescence.^274^
Monosaccharide	Galactose	Addition of galactose to maturation media improves maturation speed and total oxidative capacity.^271^
Amino acids	Taurine	Taurine is used for fat absorption, cardiomyocyte energetics, and as a pH buffer in the mitochondrial matrix for stabilization.^152,275^
	L-glutamine	L-glutamine promotes increased beating function and decreased apoptosis of cardiomyocytes.^276^
	L-carnitine	Carnitine assists in transport of fatty acids through the mitochondrial membrane.^163,277^
	Creatine	Creatine is an important temporal and spatial energy source.^275,277^
Genetic manipulation	Let-7	Let-7 enhances cardiomyocyte size, sarcomere length, contraction force, and respiratory capacity.^278^
	microRNA-1	miR-1 assists in facilitation of electrophysiological maturation.^158,279^
	microRNA-499	miR-499 promotes ventricular specification of human embryonic stem cells.^158,279^
Micropatterning	Sarcomeric alignment	Cardiomyocyte sarcomeric alignment and formation are improved after plated onto micropatterned matrices.^22,280–283^

IGF-1, insulin-like growth factor 1.

Another consideration is that the importance of preserving the matrix that the cells inhabit is also through the use of media supplements. One common approach is supplementing media with the MMP inhibitor aprotinin^171^ to prevent noncardiomyocyte cells in coculture from substantially remodeling the matrix.^172^ However, matrix degradation and production *in vivo* are in a dynamic counterbalance, and interfering MMP may subsequently impact the physiological component of the construct.^173^

### Electrical stimulation

To have a physiologically relevant cardiomyocyte tissue construct, it is important to encourage the cells to mature through electric and mechanical pacing. Pacing has been shown to improve the contractility,^174^ intracellular protein density, and transcriptome^36,175,176^ of cardiomyocytes. The biological basis for electrical pacing is to replicate a sudden charge differential between the inside and outside of a cardiomyocyte, which occurs at the beginning of an action potential. The interior of a mature human cardiomyocyte is typically polarized to −90 mV at rest, due to ion pumps keeping positive calcium ions in the sarcoplasmic reticulum or outside the cell. The initiation of contraction is accomplished through the depolarization of specialized cardiac pacemaker cells.

Cardiomyocytes in contact will spontaneously synchronize calcium channel depolarization and develop a sarcoplasmic reticulum, which may lead to rhythmic contraction within 30 days of culture.^177–181^ However, slight differences in stress and tissue geometry can alter the contraction frequency, which over time will alter tissue performance. For example, cardiac patches with a honeycombed structure permit improved cardiomyocyte alignment in comparison to disc-shaped patches, resulting in a more contractile tissue.^182^ To control the contraction differences, electrical pacing has been used, which recreates the conditions that lead to cardiomyocyte maturity in a noncontact manner. For projects intended to fabricate a surgical product, pacing is important for the integration of the resulting tissue into the native electrical pacing system in the recipient's heart.^175,183,184^ Similarly, projects intended to model the human heart must consider the development of pacemaker cells and the electric coupling of cardiomyocytes to the neuronal signal induction system found *in vivo*. Generally, electrical pacing for tissue engineering is performed by inserting an inert electrode composed of graphite or platinum into the media on either side of a tissue construct and by allowing a pulse of electrical current to pass through the tissue for a known duration (pulse width) and a known period between pulses (pulse frequency).^185,186^ The pulse generates an electric field that replicates the electrical field caused by ion movement during an action potential.^187^ Another method for electrical stimulation is the use of a salt bridge, which is the same concept but with a wet sponge separating the electrode from the target tissue.^188^ The salt bridge sponge allows current and certain ions to travel between the electrodes but prevents biomolecule aggregation on the electrodes. Both methods coordinate the tissue to repolarize and depolarize at a designated frequency, which “exercises” the construct.

As for the duration of the stimulation, a 1–2 ms pulse is sufficient for a 5 V/cm field strength over a 1 cm distance, with smaller electrical field strengths requiring larger pulse lengths.^189^ An important note is that a direct current passing between the two electrodes aggregates biomolecules on the surface of the electrodes, due to net or gross electrical charges on these molecules.^190^ A biphasic signal (a positive current that is then countered by an inverse current of equal duration, for a net current of zero) is sometimes used for stimulation to result in a net zero charge over the duration of the stimulation. However, the biphasic waveform is not physiologically accurate.^191^

Another consideration is the frequency of stimulation. Physiological heart rate ranges from 0.07 Hz (4 bpm) in diving whales to 20 Hz (1200 bpm) in hummingbirds, with humans ranging from 0.7 Hz (40 bpm) to 3.3 Hz (200 bpm). Higher stimulation frequencies can be used to investigate diseased states, representing the higher heart rate (tachycardia) required by weak tissues to pump the same amount of blood. Higher rates can also be used to “exercise” the heart to a hypertrophic state.

Stimulation intensity is often left undefined by researchers due to its complexity and the difficulty in directly measuring the intensity within the myocardium of a healthy human heart. Signal intensity for pacing is measured in units of Volts/cm (cm being the distance between electrodes) or milliamps, with relevant levels being between 0.1 and 10 V/cm^192^ and a reasonable level of continuous stimulation being 2–7 V/cm.^175,193,194^ Larger setups require larger voltages to achieve 5 V/cm, with greater distances between electrodes resulting in greater resistance and thus lower amperage and electric field strength. In terms of resistivity, the conductivity of saline solution is around 1.4 S/m or 0.7 Ω·m,^195^ giving saline a resistance equation of $Resistance\left(\Omega \right)\approx \frac{0.7\left(\Omega m\right)\cdot Distance\left(m\right)}{ElectrodeArea\left(m^{2}\right)}$. As an example, a 1 cm spacing between two 1 cm^2^ rectangular electrodes in a 24 well plate would have an end-to-end resistance of around 70 Ω per well. If 24 wells were given similar electrodes, the effective area of the electrodes would increase 24 times, and the overall resistance would be 3 Ω. Each 70 Ω well draws 70 mA from the 5 V source, which is a reasonable stimulation intensity for a 1 ms pulse.^194^

One way to quantify the safety of electrical pacing is with the use of current density, which is the number of amperes passing through a unit area (mA/cm^2^), which can be reduced by increasing the surface area of the electrodes used. Current densities below 100 mA/cm^2^ are sufficient to pace cardiomyocytes.^196^ However, a major unmet need in tissue engineering investigation is a unified protocol for pacing cardiomyocyte tissue constructs, described using quantitative measurements of construct stimulation that are interchangeable between systems.

A final consideration is the impact of electrolysis, in which a strong electric current breaks down certain chemicals into smaller molecules. One major example of electrolysis is the rapid electrodegradation of phenol, which is the active component in the pH indicator used in most forms of media.^197^ Because electrolysis occurs, it is important to carefully decide how strong of a stimulus to use and what effect the stimulus will have on the media and its content.

### Mechanical stimulation

The mechanical stimulation of heart tissue is also a significant contributor to engineered heart tissue development. The mechanical properties of the human heart change substantially as the heart matures (with stiffness increasing with age).^198^ The physical change in the stiffness of the heart is an important indicator of heart development, and the stiffness of tissue engineered cardiac constructs has an impact on construct functionality. Construct stiffness can be nondestructively quantified through physical tests of the material properties of the engineered tissue, including ultrasound and biopsies.^199–201^ Due to the conditions of cardiac tissue culturing, *in vitro* quantification of stiffness is typically accomplished by growing the tissue in specialized culture setups capable of measuring their material properties or through destructive testing.^202^ Cardiac tissue stiffens along its striations as it matures, and engineered tissues that undergo mechanical stimulation demonstrate substantial improvements in cardiac orientation.^203,204^ The mechanisms by which cardiac tissues detect mechanical stimulation involve the MAPK and ERK signaling pathways, which are modulated by stretch and cyclic stretch responsive proteins such as titin and titin-associated proteins.^142,205–207^

Mechanical stimulation has historically been performed on two-dimensional (2D) cell cultures of cardiomyocytes grown in monoculture on a flexible inert material, such as polydimethylsiloxane (PDMS). PDMS can be treated to increase the capacity of cells to adhere through a variety of techniques, including using the same plasma treatment used to create cell culture plates,^208^ functionalizing the surface with adhesive substances^209^ or washing the surface with polydopamine to increase the surface's wettability.^210^ Historically, mechanical stimulation has been achieved by modulating surface stiffness,^211,212^ stretching the material stepwise over time,^213^ or by dynamically actuating the material to simulate normal heart functionality.^214^

In recent years, 3D mechanical stimulation systems have become more common.^215–217^ These 3D systems allow for greater construct handleability, making dynamic mechanical stimulation a feasible endeavor. Dynamic mechanical stimulation achieves superior outcomes by many metrics.^214^ The quantification of dynamic mechanical stimulation has two forms, stress and strain. There are multiple forms of strain, with the most appropriate for cardiomyocytes being engineering strain which denotes the change in the length of a material in one direction. Engineering strain describes increased length (e.g., a 10% increase), whereas the strain rate of a system is the amount of strain given to a system over time (e.g., 10% over 1 s). Strain rate is important because the heart is a viscoelastic material, which makes the rate of applied strain significant in determining material behavior. Steel, for example, is not particularly viscoelastic and will snap at around the same tension whether it is stretched quickly or slowly. In contrast, biological tissue will snap easier if subjected to tension at a higher strain rate.^218^ Excessive strain rates may damage the tissue and may give incorrect measurements of cardiomyocyte contractility originating from the viscoelastic behavior of the extracellular matrix. The physiological origin of strain stimulation is the stretch experienced by cardiomyocytes during heart filling, and a strain stimulation of 10% has been used extensively for tissue engineered heart constructs.^175^

Stress, however, is the amount of tension or compression exerted on an area. A heart tissue can be stimulated with stress by giving it a set amount of tension to pull against or the construct can be stimulated by strain by stretching the tissue for a set amount. However, stress is an uncommon method of mechanical stimulation, as variability between constructs makes a unified stress stimulation protocol difficult, whereas strain is normalized to construct geometry. Both stress and strain can be used to tension a heart in one direction, which is important for proper cardiomyocyte sarcomere alignment.^219^ Some systems use a 2D stretch instead of a 1D tension, which creates an isotropic force that produces a radial cardiomyocyte orientation, as seen at the apex of the heart.^220^

Both mechanical and electrical stimulation can be used in conjunction to produce excellent systems capable of stimulating tissue engineered heart constructs in a physiologically applicable way.^175^

## Engineered Cardiac Construct Function and Molecular Assessment

A final consideration is the analysis of tissue engineered heart constructs. The primary methods of analyzing a tissue engineered heart construct include electrophysiological, biochemical, and morphological analysis. The intended purpose of the construct greatly alters the importance of the various parameters outlined in the following section. For instance, modeling-based projects may attempt to maximize the transcriptomic and proteomic applicability of their construct, with little regard for the contraction strength of the construct. In contrast, surgical product-based projects may hope to maximize the contractility of their constructs and focus their transcriptome analysis on conduction and structural biomarkers.

Contractility analysis is a nondestructive test that is unique to each construct, with 2D constructs typically involving video analysis of the monolayer^115^ or specifically designed bioreactors capable of transducing contraction into a force value.^221^ 3D constructs allow for direct measurement of contraction using force sensors or calibrated materials for force quantification.^67,215,216^ Contractility analysis can be used to demonstrate the maturation of the heart tissue by quantifying frequency, contraction duration and amplitude variance, and rate of contraction and relaxation. The action potential can also be analyzed nondestructively using patch clamp or calcium-sensitive dyes.^122,222,223^ Another method of analyzing the action potential of cardiac constructs is through voltage sensitive dyes and gene-encoded voltage indicators. With the use of membrane potential dyes, physiological changes such as muscle contraction and cell signaling can be more accurately measured and analyzed.^224,225^ Unfortunately, the unique physical shapes of different constructs make contractility data difficult to compare between systems, although 3D systems can divide construct force by construct cross-sectional area to quantify tissue level contraction strength. Destructive tests that isolate the contractile machinery allow for a standardized comparison between cardiomyocytes from different systems.^226^

Proteomic and transcriptomic analysis are also highly important in the quantification of heart construct functionality. Several biomarkers can be used to quantifiably compare constructs, establish a measurement of maturity (*MYH6* levels compared to *MYH7*), cell population identity (*CD31* for endothelial cells, *PDGRFA* for fibroblasts), and to assess for cardiomyocyte subtype heterogeneity (*MYL2* for ventricular, *SLN* for atrial). Quantification of biomarkers allows for the systematic improvement of constructs into progressively more physiologically applicable models.

Furthermore, given that CRISPR/Cas9 technology has become more widespread, genetic manipulation of iPSCs is now becoming routine. It is now possible to directly compare cardiomyocytes from an iPSC line harboring cardiomyopathy causing mutations in direct comparison with isogenic corrected hiPSC-CMs. In addition, CRISPR/Cas9 can be used to fluorescently tag various cardiac proteins such as titin so contractile analysis can be performed easily.^227^ Furthermore, the use of CRISPRi and CRISPRa within iPSCs now presents the opportunity to turn on or off genes being studied either during differentiation or to assess end states of disease.^228–230^

Morphological analysis is primarily performed through the use of histology techniques such as immunohistochemistry to identify the location and concentration of substances of interest.^231^ Hematoxylin and eosin stains are used to visualize the extracellular matrix and cell density inexpensively, while fluorescent antibodies commonly used to inspect sarcomeric integrity and alignment include cardiac troponin T (*TNNT2*), actin (*ACTB*), myomesin (*MYOM1*), and actinin (*ACTN1* and *ACTN2)*.

## Conclusion

The field of heart tissue engineered is exciting, relevant, and has the potential to revolutionize drug screening, cardiovascular surgical techniques, and eventually even human heart transplants. The general trends in cardiac tissue engineering are dependent on the project type. For modeling-based projects, the general trends are to develop techniques for differentiating a broader array of cell types, generate constructs with specific numbers of different cell types placed in a particular spatial distribution, and to improve the applicability of construct behavior using more relevant electrical and mechanical pacing techniques. For surgical product-based projects, the general trends are to develop more sophisticated and tuned matrix geometries and compositions, cell type mixes with greater contractility, and pacing techniques that achieve stronger contractions and capacity for electro-coupling with the existent heart. However, both types of projects suffer from a lack of standardization.

The future of cardiac tissue engineering must include a greater standardization of techniques to facilitate more rapid technology transfer between research groups. A standardized protocol for electrical and mechanical stimulation that takes electrode and construct geometry into account is needed, as is a standardized media composition schedule that optimizes cardiomyocyte development and maturation. Furthermore, a standardization of input factors and analytes, including cell count, maturity markers, cell origin, and matrix density, would help to isolate the influence of controlled variables. As for the future of tissue engineering projects, there is a substantial unmet need for a commercially available, electrically and mechanically paced, chambered, and macroscopically 3D construct generation system. Such a system would be able to include other important forms of stimulation for the heart, including surface shear and spatially separated cell layers.

While there have been significant advancements in assembling and functionally assessing cardiac constructs, it is still not possible to perfectly mimic the human cardiac cell populations (cardiac fibroblasts are derived from multiple developmental cellular lineages), develop a stable and functional tissue matrix,^232^ model chamber specific spatial pathophysiologies (fatty-fibro infiltration), establish robust and synchronous cardiac contraction,^233^ and engineer vascularized constructs at the thickness of the adult heart.^234^ The tissue engineering field is continuously evolving as the need to understand the complexities of cardiovascular diseases and disorders.^235^ Stepwise progress will eventually lead to a robust platform to understand and model various cardiomyopathies with the goal to create a functional engineered heart designed for transplantation.^236,237^ Although this is an active field of research, the future of engineered heart models is promising.^238^
